# Energy Efficient All-Electric-Field-Controlled Multiferroic Magnetic Domain-Wall Logic

**DOI:** 10.1021/acs.nanolett.3c00707

**Published:** 2023-07-19

**Authors:** Xin Li, Hanuman Singh, Yi Bao, Qiang Luo, Shihao Li, Jyotirmoy Chatterjee, Maite Goiriena-Goikoetxea, Zhuyun Xiao, Nobumichi Tamura, Rob N. Candler, Long You, Jeff Bokor, Jeongmin Hong

**Affiliations:** † School of Sciences, Hubei University of Technology, Wuhan 430068, China; ‡ School of Integrated Circuits, Huazhong University of Science and Technology, Wuhan 430074, China; § EECS, UC Berkeley, Berkeley, California 94720, United States; ∥ IMEC, Leuven 3001, Belgium; ⊥ Department of Electricity and Electronics, University of the Basque Country (UPV/EHU), Leioa 48940, Spain; # Department of Electrical and Computer Engineering, UCLA, Los Angeles, California 90095, United States; ∇ Advanced Light Source, Lawrence Berkeley National Lab, Berkeley, California 94720, United States

**Keywords:** Multiferroic coupling, electric-field-controlled strain, magnetic domain wall, Boolean logic, energy efficient device

## Abstract

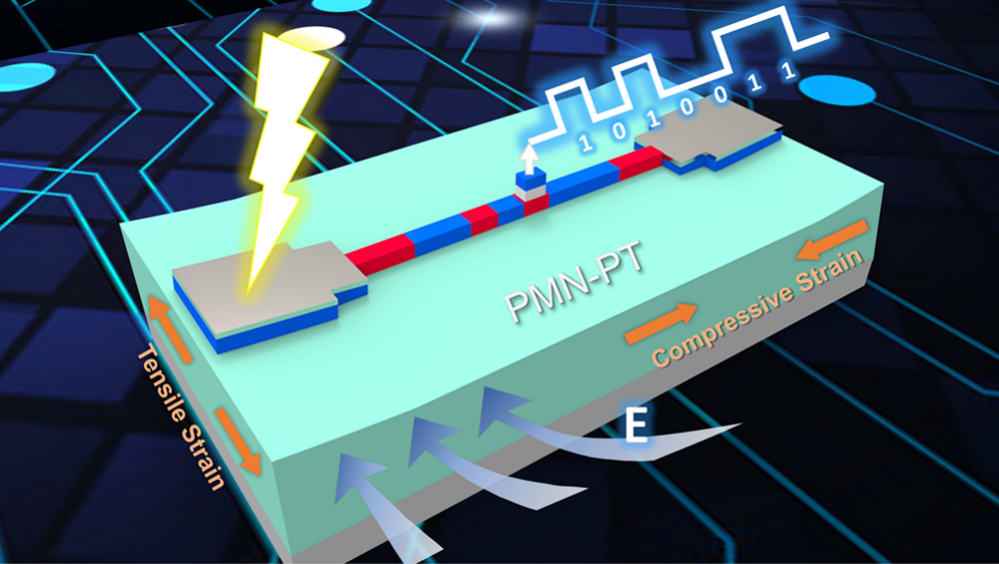

Magnetic domain wall (DW)-based logic devices offer numerous opportunities for emerging electronics applications allowing superior performance characteristics such as fast motion, high density, and nonvolatility to process information. However, these devices rely on an external magnetic field, which limits their implementation; this is particularly problematic in large-scale applications. Multiferroic systems consisting of a piezoelectric substrate coupled with ferromagnets provide a potential solution that provides the possibility of controlling magnetization through an electric field via magnetoelastic coupling. Strain-induced magnetization anisotropy tilting can influence the DW motion in a controllable way. We demonstrate a method to perform all-electrical logic operations using such a system. Ferromagnetic coupling between neighboring magnetic domains induced by the electric-field-controlled strain has been exploited to promote noncollinear spin alignment, which is used for realizing essential building blocks, including DW generation, propagation, and pinning, in all implementations of Boolean logic, which will pave the way for scalable memory-in-logic applications.

The energy consumption of a spin device from magnetoelastic materials should be significantly smaller than those of CMOS transistor-based logic devices.^1,2^ For this reason, the research on magnetic domain wall (DW)-based devices is of great interest beyond CMOS devices. In a DW structure, the magnetizations of the two domains point in opposite directions.^3−12^ DWs in ferromagnetic (FM) nanostructures^3−5^ have shown a variety of potential applications in spintronic devices, including DW logic gates^6,7^ and racetrack memory.^8−12^ In such a device, DW is used as bits of information that can be controlled with an electric field.^13^ The ability to precisely control and manipulate the DW with an electric field is important for designing a low-power, high-density circuit device.^12^ DW is usually controlled using an external magnetic field,^15^ but the control of the DW propagation with a magnetic field limits the density and the energy efficiency of the device.^14^ Another method to control DW is to use spin-polarized current to switch the magnetization in magnetic thin films through spin transfer torque (STT)^16−21^ or spin orbit torque (SOT).^22−25^

There have been many efforts to improve efficiency, high-speed switching, and device density.^46^ The device concepts that have recently emerged using electric field control of magnetization switching^26−28^ provide an alternative route to achieve fast and high-density information storage with very low energy consumption.^29^ A multiferroic heterostructure composed of FM and ferroelectric (FE) materials is well-suited for high-speed, low-power, and ultradense device structures.^29−34^ So far, there have been three main methods available for the electric field control of magnetism in FM/FE multiferroic heterostructures, including (i) strain-mediated magnetoelastic coupling with the piezostrain of the FE transferred to the FM layer,^32,35^ (ii) exchange bias-mediated interaction,^25,36^ and (iii) manipulation of charge carrier density.^37,38^ Among them, the electric field control of magnetism through strain-mediated coupling in FM/FE multiferroic heterostructures has become a hot topic due to the availability of a variety of room-temperature FM and FE materials and the remarkable magnetoelectric effects.^35^ The strain is induced by applying an electric field to the FE layer via the piezoelectric effect, and the induced strain is then transferred to the FM layer, altering the magnetization via magnetostriction. Among the piezoelectric materials, lead manganese niobate-lead titanate, i.e., (1–*x*)Pb(Mn_1/3_Nb_2/3_)O_3_-*x*PbTiO_3_ (*x* = 0.3), PMN-PT, is a common material because the FE phase in multiferroic magnetoelectric heterostructures due to the ultrahigh in-plane anisotropic piezoelectric response in both single crystals and epitaxial thick films can effectively modulate the magnetic properties.^39−41^ The induced in-plane anisotropic localized ferroelastic strain can induce an easy magnetic axis in the contacted magnetic thin film,^42^ which in turn enables locally different manipulation of the domain structure.

In this work, we demonstrate a specially patterned, structured multiferroic Ni/PMN-PT heterojunction logic device based on magnetic DW movement. Numerical and micromagnetic simulations performed by COMSOL and the object-oriented micromagnetic framework (OOMMF),^43^ as well as experiments, have been demonstrated to investigate the effect of electric field on DW at the generation mode, propagation mode, and termination mode of DW. Based on this, two different device structures are proposed and verified through micromagnetic simulation, which could realize XOR/XNOR and OR/NAND logic functions, respectively.

## Properties of Ferromagnet on PMN-PT

**Figure 1:**
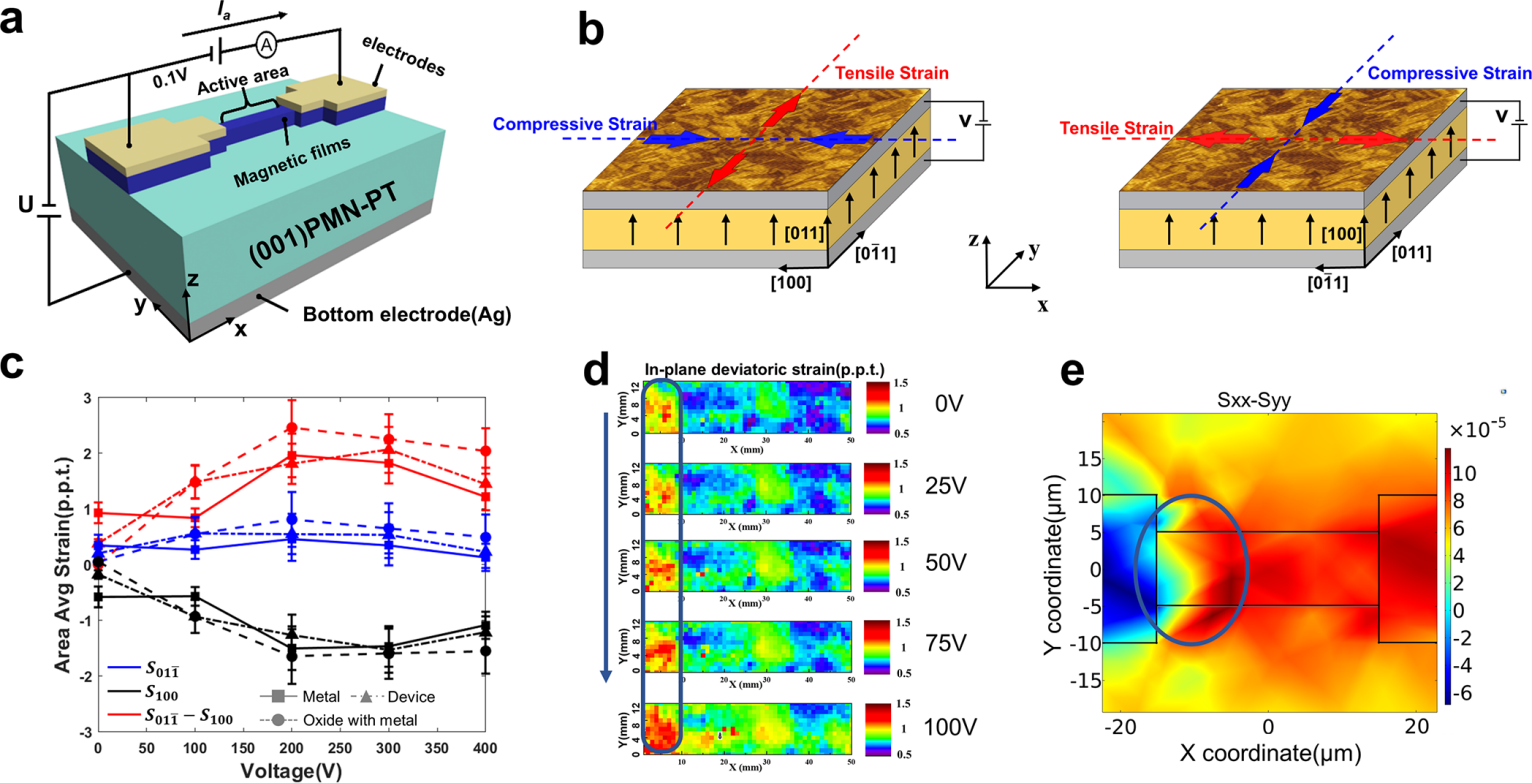
(a) Schematic of the device structure for the measuring system. (b) Strain direction on [011]- and [100]-polarized PMN-PT, under a vertical upward electric field. (c) Area average strain of metal, device, and oxide with metal in [100], [011] in-plane direction and the difference between them. (d) Experimental results of the active area under different voltages. (e) Simulation results of the active area under 400 V (above the saturation voltage).

First, we investigated the mechanical and magnetic properties of a Ni thin film on the (011)-oriented PMN-PT substrate under the action of an electric field. The ferromagnetic nanowire structure is deposited on top of the PMN-PT substrates, as shown in Figure 1a. Two top electrodes are deposited on both ends of the magnetic thin film, and the region without top electrode deposition in the middle part is called the active area. A double-sided, polished, 500 *μm* thick piezoelectric PMN-PT single crystal with both the top and bottom surfaces covered by Pt electrodes was studied. Before depositing the Ni thin film on top of it, the PMN-PT substrate is electrically prepoled in the [011] and [100] directions, respectively, with the polarization pointing “up”. Under the action of the vertical upward electric field, the polarization in the [011] direction of PMN-PT will generate compressive strain in the [100] direction and tensile strain in the [011̅] direction, while the polarization in the [100] direction will generate compressive and tensile strain in the [011] and [011̅] directions, respectively, as also shown in Figure 1b (left and right).

Microscale strain response is characterized through X-ray microdiffraction at Advanced Light Source (ALS) on the aforementioned sample, in which the strain is induced by stepping the electric field from 0 to 0.8 MV/m. In Figure 1c, the area average strains of the metal, device, and oxide with metal in [100] and [011̅] in-plane directions and the difference between them are presented. The strain data are tested under different substrate voltages. When an electric field is applied along the [011] direction, the [100] crystallographic direction produces a compressive strain (*S*_100_ < 0), and the [011̅] crystallographic direction produces a tensile strain (*S*_011̅_)> 0). It can be seen that the top film thickness has a very small effect on the substrate strain, and the average strain on different film stacks is almost equal.

X-ray microdiffraction (Figure 1d) and simulation (Figure 1e) results of the active area in the device under the applied voltage are demonstrated. The experimental results show that the strain intensity is higher in the parts that are closed to the electrode of the active region, and this rises as the applied voltage increases. The simulation results obtained from COMSOL confirmed that the strain distributed nonuniformly on PMN-PT substrate, which is consistent with the experimental test data. Nonuniform distribution of strain has been pointed out by the dark blue circle in Figures 1d and 1e.

## DW Propagation

**Figure 2:**
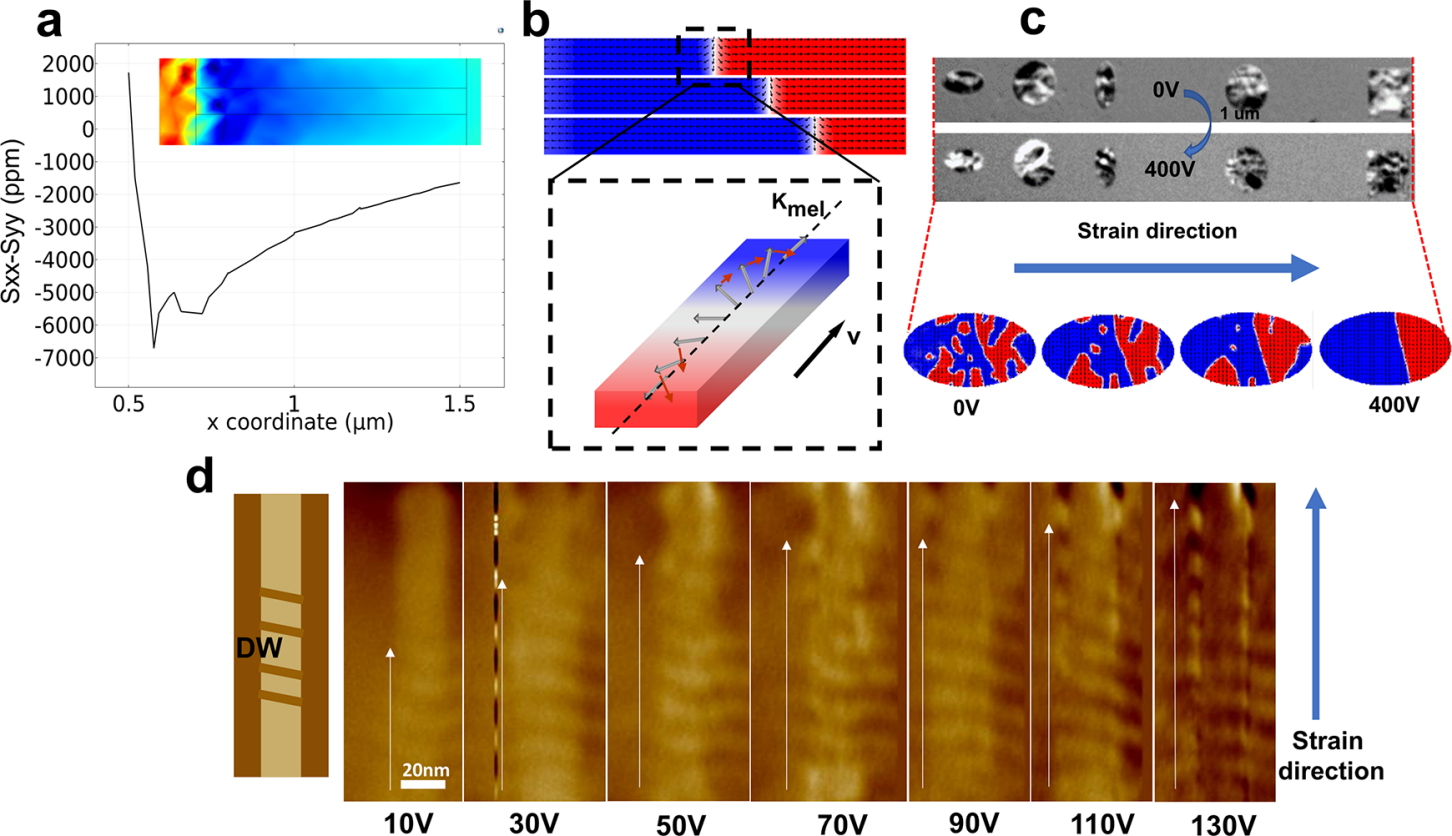
(a) In-plane strain (ε_*xx*_ – ε_*yy*_) distribution of FM nanowire along the *x* direction. (b) Propagation and schematic of the Neel DW under strain distribution. (c) PEEM images and micromagnetic simulation results of DW propagation under 0 and 400 V voltage. Blue arrow shows the generation of strain. (d) MFM images of DW propagation. Schematic of the measurements and domain wall locations are shown on the left.

Ni with in-plane magnetic anisotropy is grown on a [011]-oriented PMN-PT single crystal. The in-plane strain (ε_*xx*_ – ε_*yy*_) distribution of an FM nanowire along the *x* direction is presented in Figure 2a. The calculation was performed with the COMSOL Multiphysics package, with a 400 V vertical voltage applied through the electrodes. A larger negative in-plane strain occurs near the top electrode, where the voltage is applied. The longer the distance away from the top electrode cases is, the smaller the absolute value of the negative in-plane strain is. The in-plane strain distribution at the interface between the PMN-PT and FM thin film is shown in the inset image of Figure 2a. It can be found that the interface exhibits an overall in-plane strain, which is tensile along the [100] direction and compressive along the [011] direction. It is noted that not far from the top electrode a large negative in-plane strain is generated.

To simulate the situation where the aforementioned strain acts on a DW in this device design, micromagnetic simulations are performed on a wire geometry. OOMMF magneto-elastic energy module^43^ is performed to induce magneto-elastic energy. Strain distribution is converted to a spatially correlated uniaxial anisotropy-like magnetoelastic energy using the magnetoelastic coupling constant, in which *B*_1_ = 6.2 × 10^7^ erg/cm^3^ and *B*_2_ = 9.0 × 10^7^ erg/cm^3^, consistent with the corresponding constant in Ni. Detailed simulation settings are described in the Methods Summary. Neel magnetic DWs will propagate in FM nanowires under the strain distribution analyzed before. A schematic of Neel DW (Figure 2b) can help explain the mechanism of DW propagation. The in-plane negative internal strain mentioned in Figure 1a induces an easy magnetization axis, *K*_mel_, along the longitudinal direction of the nanowire. The magnetic moment in the Neel DW is affected by the torque shown in the graph. Due to the nonuniform distribution of the in-plane strain, the magnetic moment of the magnetic DW near the top electrode, to which the voltage is applied, is subjected to a larger moment than that of the end which is far from the top electrode. Therefore, under the combined action of this asymmetric torque and exchange effect, the magnetic DW will propagate away from the top electrode on which the voltage is applied.

Except for the nanowire structure, DW propagation in the FM layer with different shapes is investigated, through X-ray magnetic circular dichroism-photoemission electron microscopy (XMCD-PEEM, shortened as PEEM) measurements, to find out other effects such as the DW pinning and the presence of a vortex structure. Figure 2c shows the PEEM images that demonstrate DW propagation under 0 and 400 V, with an electric field perpendicular to the film plane and pointing up, in FM films of different shapes. DWs were propagated through the strain direction in all different shapes. For the double check, micromagnetic simulation was performed on an FM layer with elliptical shape as shown in Figure 2d. The device is randomly magnetized at the initial state, and the DW is generated and then propagates after the electric field is applied. Figure 2e shows magnetic force microscopy (MFM) results about DW propagation on an FM layer in a strip shape. From 0 to 200 V, DW propagation was observed in this structure. Propagation of DW can be matched through both of these two ways: PEEM and MFM.

## DW Creation and Termination Mechanism Analysis

**Figure 3:**
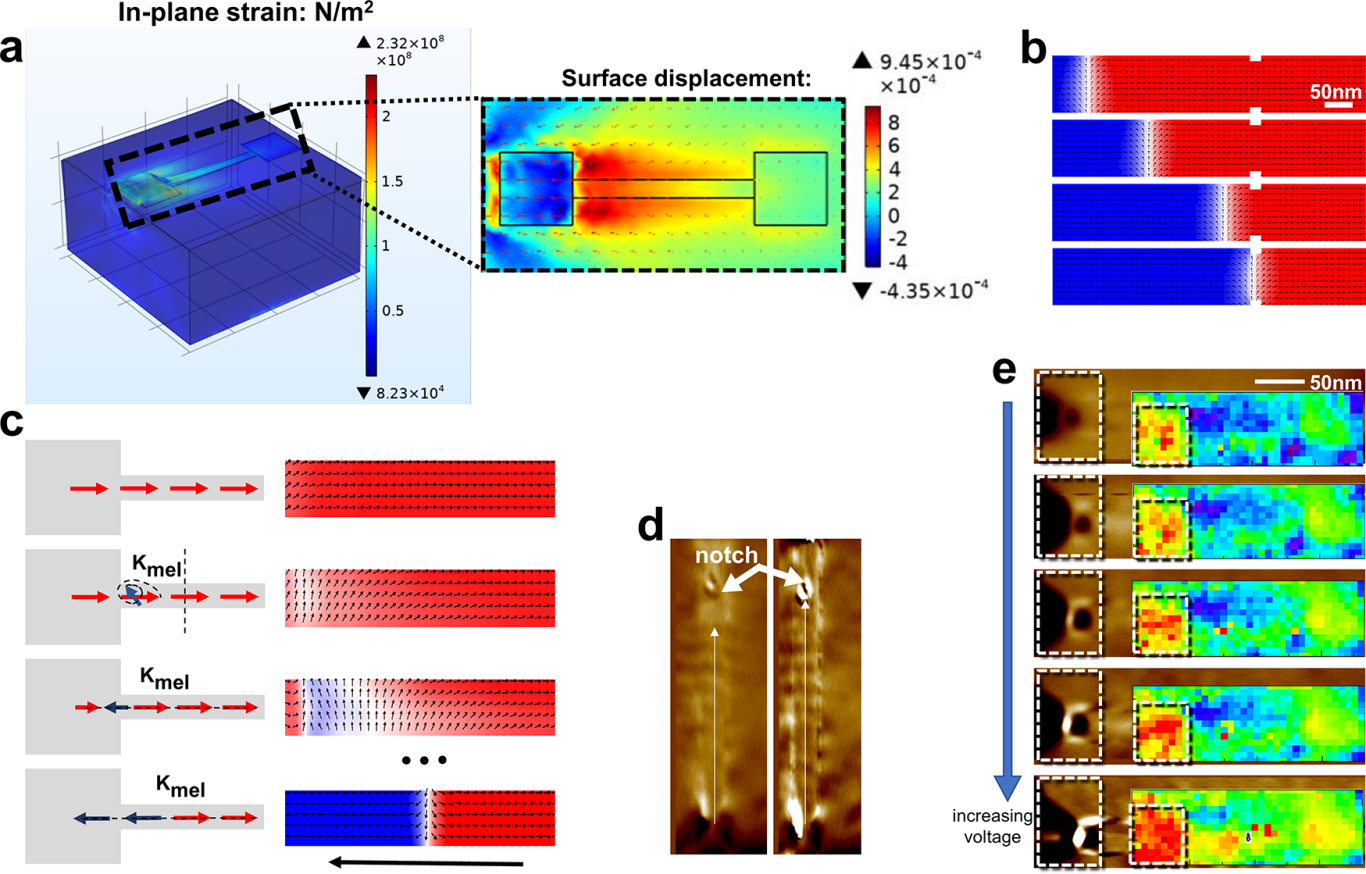
(a) Deformation and in-plane strain distribution of the device after applying electric field. (b) Micromagnetic simulation of the DW termination at the notch. (c) Schematic of the nanowire showing precession under the strain-induced magnetic easy axis. (d) MFM images show that, under voltage of 30 V (top) and 200 V (bottom), the DW is created, propagated, and terminated. (e) X-ray microdiffraction and MFM confirm the generation and precession of DW under the magnetoelastic energy in nanowire.

First, we investigated the generation of the magnetic DW. By changing the polarity of the electric field acting on the substrate, tensile strain is generated in the [100] direction, and compressive strain is generated in the [01̅1] direction under a pointing down electric field. From the stress distribution, deformation and in-plane strain distribution on the device after applying an electrical field are shown in Figure 3a. Due to the different sizes of edge and middle regions, the amplified strain is generated and then domain generation is made from a ferromagnet. It can be found that a large positive in-plane strain (*S*_*xx*_*- S*_*yy*_ > 0) is generated near the top electrode where the voltage is applied, which is transferred to the Ni layer and induces a magnetic easy axis along the [011] direction. The boundary pinning effect of notches during domain propagation to achieve DW termination is also experimentally investigated and simulated. Micromagnetic simulation results of DW termination are shown in Figure 3b. Under the action of an electric field, it can be found that the relaxed magnetic DW continuously propagates and finally terminates at the notch.

Figure 3c shows magnetic precession of the nanowire, which verifies that the induced compressive strain imparts an easy magnetic axis due to the inverse magnetostrictive effect in a negative magnetostrictive material, such as Ni. The resulting easy axis favors a two-domain state, where the magnetization in the two domains is aligned along the new magnetic easy axis, finally in an antiparallel fashion. A reverse magnetic domain larger than 90° can be generated at a certain moment during the precession. The bottom image of Figure 3c shows the full scenario of the structure, from creation to termination of the DW at the notch, which has been confirmed through MFM measurements as shown in Figure 3d. The nanowire device is uniformly magnetized before the electric field was applied. A full 180° rotation of magnetization direction and Neel DW can be achieved by changing the electric field polarity at this certain moment. DW generation has also been confirmed by MFM methods, as shown in Figure 3e. Voltage-induced inhomogeneous strain in this region tested through X-ray microdiffraction is shown in the insets, which helps explain the reason for DW creation.

## Logic Operation

**Figure 4:**
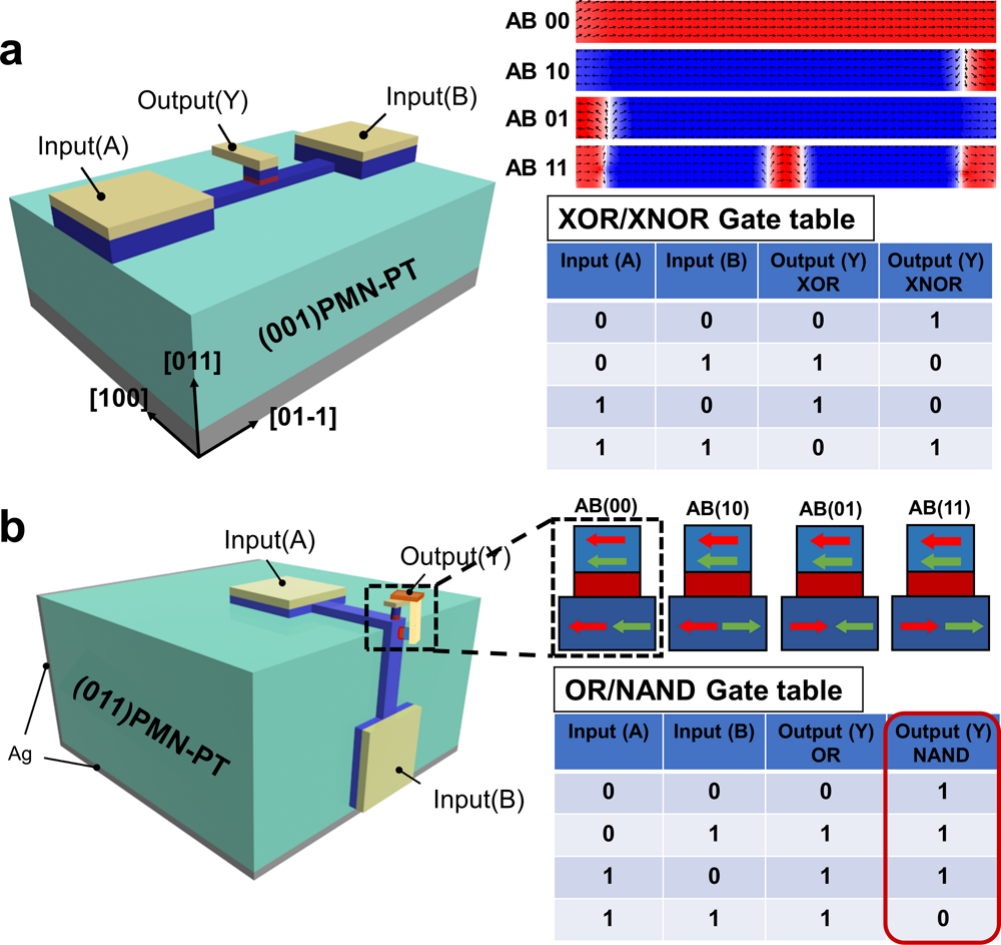
(a) Structure and principle of the XOR/XNOR logic gate. (b) Structure and principle of the OR and NAND logic gate.

Based on the above-investigated mechanism and method of the generation, propagation, and termination of magnetic DWs, we propose two structures for the demonstration of XOR, XNOR, OR, and NAND logic gate operation. Figure 4a shows how XOR and XNOR logic gates can be built with a simplified structure. Ni thin film and two top electrodes are stacked on the [011]-oriented PMN-PT substrate with a bottom electrode. The magnetic easy axis of the FM nanowires is in the [011] direction. The two electrodes are defined as input A and input B, respectively. A half magnetic tunnel junction (MTJ) structure is stacked above the middle of the nanowire, which is used to detect the magnetization below the half MTJ stack, in which the resistance state of the whole MTJ stack is defined as the output Y. The FM layer in the half-MTJ structure and the nanowire act as the fixed and free layers of the whole MTJ, respectively. The logic states of input A and input B are recorded as “1” or “0” when the input end is connected to high voltage or ground. It is worth mentioning that a reversed short voltage pulse needs to be applied to generate reversed magnetic domains before the logic operation. When the input combination AB is “01” or “10”, the magnetic DW propagates until the nonuniform strain is not enough to overcome the depinning field of this static DW structure to continue driving the magnetic DW, and then stops. When the input combination AB is “11”, strain in the nanowire reaches a minimum value at the middle part, so the magnetic DWs at both ends of the nanowire stop propagating before reaching the middle part. Final magnetization distribution in the nanowire under different input combinations is simulated through OOMMF. The low/high-resistance state of the aforementioned MTJ stack is recorded as logic “1” or “0” in the output end. If the magnetization direction of the fixed layer is initialized to be opposite the magnetization direction of the nanowire, a logical XOR function can be achieved, and vice versa, a logical XNOR function can be achieved. The truth table is also listed.

OR and NAND logic gate structures are also built (Figure 4b). FM layers and top electrodes are stacked on the [011] and [100] planes, respectively, where the electrodes are used as input A/B. Two-half MTJ structures are stacked above the ferromagnetic layer on the [011] and [100] planes near the junction. For the output Y, which is composed of the two aforementioned half-MTJs, its magnetization state can be deduced, as shown in the schematic. The boundary between the two planes can also serve as a notch to terminate the propagation of the magnetic DW. The final magnetization of the output under different input combinations can be deduced, and the truth table is also listed. In this way, OR and NAND logic functions could also be implemented.

We proposed a special-structured multiferroic Ni/PMN-PT heterojunction device that is capable of performing all-electric logic operations using a DW racetrack. An electric field can control the local magnetic domain to realize a 180° magnetization switching. Ferroic coupling between neighboring magnetic domains can be induced by the electric field-controlled strain, which promotes noncollinear spin alignment, to realize DW generation (input), propagation, and pinning (output). To achieve this capability, a new type of all-electric, field-controlled spin logic device is designed. We implemented essential basic building blocks in functional logic, such as Boolean logic operations, including XOR/XNOR, OR, and NAND, by demonstrating electrical control on a single crystalline PMN-PT through multiphysics and micromagnetic simulation. The DWs are controlled by biaxial strain generated with the piezoelectric substrate by applying an electric field of 0.8 MV/m. If a thin-film piezoelectric material with a thickness of 100 nm is used to produce this similar strain, we can achieve this electric field by only applying 80 mV voltage.^44,45^ The result paves the way for scalable all-electric magnetic memory-in-logic applications with low power consumption.

## Methods Summary

### Device Fabrication

Ni films with thickness of 40 nm were deposited on the PMN-PT substrate by radio frequency (RF) sputtering at room temperature. The RF power was 20 W. The argon gas pressure was 0.2 Pa, and the sputtering time was 12 min. Then a 5 nm Pt layer was deposited on top of Ni using direct current sputtering. The device patterns with length = 200 nm and width = 20 nm were patterned through electron beam lithography and lift-off processes. The notch is about 2 nm × 2 nm, located on both sides of the output region. The insulating layer Al_2_O_3_ (30 nm) was grown by atomic layer deposition at 200 °C. Then the Al_2_O_3_ film on the electrode areas of Ni was etched.

### VSM

The conventional volume averaging magnetometry measurements were performed by using a VSM 7400 from Lake Shore Cryotronics Inc. with a 3.1 T electromagnet. The sample was mounted on a quartz holder. The magnetic moment was measured and averaged.

### SPM/MFM

Scanning probe microscopy (SPM) was performed in noncontact mode by using a Bruker-Nano AFM system. The MFM measurements were conducted in dynamic lift mode with a lift distance of 30 nm. The dynamics were measured in the presence of a magnetic field by sweeping the field range. In this type of crystal structure, the domain is randomly oriented, and the sizes of the domain structures are very large (more than a micron).

### X-ray Diffraction

Laue X-ray microdiffraction was used to investigate the elastic strain distribution on the heterojunction device. During microdiffraction scanning, individual diffraction patterns are collected step by step from grid points to provide information about lattice strain and crystal orientation. The electrically induced deviatoric strain is calculated for each step by taking the difference between the extracted strain at a nonzero voltage and at a zero voltage. This is represented by a 10 × 10 μm^2^ pixel in the constructed 2D strain maps. The Laue method is adopted to experimentally measure the in-plane deviatoric strain components, ε′_*xx*_ and ε′_*yy*_, as they are the main components driving in-plane magnetization rotation or switching.

### PEEM

The magnetic state is imaged by X-ray magnetic circular dichroism-photoemission electron microscopy (XMCD-PEEM). Exploiting the probe depth of approximately 5 nm and the elemental sensitivity of X-ray absorption at the Ni L_3_-edges, we are able to separately image the magnetic state in each magnetic layer and compare them with each other. Accordingly, from now on, we present only the XMCD-PEEM images referring to the Co layer for simplicity unless otherwise noted.

### COMSOL Simulation

From COMSOL, coupling in the Multiphysics field could be used to simulate the coupling effect between the piezoelectric substrate and FM thin film in a composite multiferroic heterostructure system. Physics modules, including the Structural Mechanics Module, Piezoelectric Module, and Magnetostrictive Module, should be added. In the Multiphysics field, the piezoelectric effect and magnetostrictive effect are considered in the piezoelectric substrate and magnetic thin film, respectively. The piezoelectric substrate is in close contact with the magnetic thin film. [011] PMN-PT is set as piezoelectric material (2 μm × 2 μm × 1 μm); FM nanowire (1 nm × 100 nm × 1000 nm) is set on the substrate with two electrodes (400 μm × 400 μm × 1 nm), with the material setting as Ni. FM nanowire is put in the middle of the piezoelectric substrate. A voltage is applied to the piezoelectric substrate to ensure the generation of strain and transfer it to the magnetic thin film.

### Micromagnetic Simulation

Magnetoelastic energy is calculated in the OOMMF with the help of YY_FixedMEL: magnetoelastic term, based on the displacement field, in the form of Oxs_VectorField, which corresponds to the strain distribution in COMSOL. It is noted that the mesh cell setting in the OOMMF should correspond exactly with the displacement field data exported from COMSOL, and the cell size should be smaller than the diffusion length. The nanowire (1 μm × 100 nm × 1 nm) is simulated by the finite element method (FEM) with a mesh size of 5 nm × 5 nm × 5 nm and a lateral notch size of 20 nm × 10 nm. Saturated magnetization *M*_*S*_ = 0.66 MA/m. Gilbert damping constant α = 0.045. In-plane magnetic anisotropy is simulated by using a uniaxial anisotropy constant with *Ku* = 10 kJ/m^3^.

## Data Availability

All data are available upon request.
